# Problematic Social Network Use: Structure and Assessment

**DOI:** 10.1007/s11469-021-00711-y

**Published:** 2021-12-02

**Authors:** Covadonga González-Nuevo, Marcelino Cuesta, Álvaro Postigo, Álvaro Menéndez-Aller, José Muñiz

**Affiliations:** 1 University of Oviedo, Oviedo, Spain; 2 University of Nebrija, Madrid, Spain

**Keywords:** Social networks, Problematic social media use, Social comparison, Addictive social media use

## Abstract

Using social networks (SNs) inappropriately can lead to psychological problems. The objective of this study was to develop a new measuring instrument of problematic use of SNs. The sample comprised 1003 participants over 18 years old (*M* = 42.33; *SD* = 14.32). Exploratory factor analysis was performed with a randomly selected 30% of the sample, and confirmatory factor analysis with the remaining 70%. The reliability of the instrument was estimated, and evidence of validity in relation to the variables—anxiety, depression and satisfaction with life—was obtained. The new scale demonstrated a two-dimensional structure (GFI =0.99; RMSEA= 0.06), with one factor of negative social comparison (*α* = 0.94) and another of addictive consequences (*α* = 0.91). Clear evidence of validity related to other variables was found. The new scale demonstrated good psychometric properties. The advantage of this questionnaire is that it assesses not only excessive use but also social comparison through SNs.

Time spent in social networks (SNs) has been linked to various psychological problems such as depression, anxiety and psychological well-being (Frost & Rickwood, 2017; Hussain & Griffiths, 2021). However, in order to clarify this relationship, it is necessary to determine which behaviours in SNs are truly problematic, as certain activities in SNs may not be problematic but positive, such as using SNs as a form of social support (Gilmour et al., 2020).

Various terms have been used to refer to maladaptive SNs use, such as problematic social media use (Bányai et al., 2017), social media disorder (Van Den Eijnden et al., 2016) and Facebook addiction (Andreassen et al., 2012). Despite this broad range of terminology, there are two main approaches for evaluating the problematic use of SNs, each one related to the two existing models for problematic internet use (Matute, 2016). One approach conceives problematic SNs use as a problem of addiction, and thus focuses on symptoms usually present in behavioural addiction (Allahverdi, 2021; Andreassen et al., 2012; Blanca & Bendayan, 2018; Van Den Eijnden et al., 2016). The other approach applies Caplan’s (2010) model of problematic internet use to SNs.

Studies based on Caplan’s (2010) model not only address typical symptoms of behavioural addiction, such as mood-related and negative consequences, but also include other traits which would denote problematic use (Marino et al., 2016). One such trait is particularly important in problematic SNs use and could be assessed alongside addictive traits and is named negative social comparison (Verduyn et al., 2020). Negative social comparison on SNs, defined as comparisons that make a person feel inferior to others on SNs, has been related to negative mental health consequences (Appel et al., 2015). To the best of our knowledge, no instruments have been developed so far that evaluate this specific behaviour on SNs.

Social comparison was defined by Festinger (1954) as the tendency to use other people as sources of information to examine one’s own abilities and ways of behaving, thinking, or feeling. These comparisons, which are innate in humans, are intensified on SNs due to the almost limitless content available (Perloff, 2014). This comparison can have positive or negative consequences, depending on which one compares oneself with. There are two possibilities: either the person compares themselves with someone they consider inferior (downward comparison) and their self-esteem improves or they compare themselves with someone considered superior (upward comparison), which is a common situation on SNs and is known to have negative effects on a person’s mental health (De Lenne et al., 2018; De Vries et al., 2018; Luong et al., 2019; Verduyn et al., 2020).

The assessment of social comparison on SNs has mostly depended on unvalidated questionnaires. Two types of questionnaires have been used without the corresponding psychometric analysis. On the one hand, there are unvalidated questionnaires that have been constructed for a specific study, such as the questionnaire by Lee (2014), in which the author makes no mention of factorial structure or reliability. However, it has continued to be used in subsequent studies (De Vries & Kühne, 2015; Schmuck et al., 2019). Another example of an “ad hoc” questionnaire about SNs comparison is in Cramer et al. (2016), in which only reliability was addressed. On the other hand, researchers have modified questionnaires aimed at evaluating social comparison in other contexts to assess social comparison on SNs. For example, a common practice to adapt them to the SNs context is to change the content of the items, mainly, by adding the word “Facebook”. For instance, Liu et al. (2017) adapted an instrument aimed at assessing comparison in day-to-day life, the Iowa Netherlands Comparison Measure (Gibbons & Buunk, 1999), to the context of SNs.

The most commonly studied psychological problems related to problematic SNs use are anxiety, depression and psychological well-being, with gambling and cyberbullying as other less frequently related issues (Feijóo et al., 2021). Problematic use, understood as a purely addictive problem, has been associated with higher levels of depression and anxiety (Barbar et al., 2021; Keles et al., 2020; Malaeb et al., 2021; Seabrook et al., 2016 ; Youssef et al., 2021), stress (Hussain & Griffiths, 2018) and poorer psychological well-being (Marino et al., 2018b). Problematic SN use, when considered a context for negative social comparison, has also been associated with greater levels of depression (Yoon et al., 2019) and anxiety-depressive symptoms (Schmuck et al., 2019), as well as with lower levels of psychological well-being (Arias-de la Torre et al., 2020; Huang, 2017). Recent meta-analyses have also studied problematic SNs use by combining both types of uses, addictive and comparative, which they have found to be both positively related to depression (Vahedi & Zannella, 2021; Yoon et al., 2019).

In summary, there seems to be a relationship between the development of psychological disorders and the problematic use of SNs. The strength of this relationship, however, largely depends on how the use of SNs is defined, and therefore measured. To date, research has addressed this relationship by understanding the problematic use of SNs as a purely addictive problem. Our study aims to bring a broader approach by developing a tool that also assesses social comparison on SNs, which is not only a fundamental characteristic of SNs but it is also completely different to the social comparison that takes place in other contexts. This questionnaire, meant to be used with any SNs, is the first one in the Spanish context. Therefore, our main objective in this study is the development and validation of a measuring instrument that will allow standardized, rigorous measurement of problematic SNs use. In order to do that, we will perform the appropriate psychometric analyses to provide evidence of item quality and relationship with other variables, along with reliability and dimensionality.

## Method

### Participants

The sample was initially composed of 1,059 participants from the Spanish general population over 18 years old. The final sample was reduced to 1,003 following the removal of 5.29% of the sample for having more than two incorrect answers in the attentional control scale (described in more detail in the “Instruments” section). The participants were aged between 18 and 83 years old (*M* = 42.33; *SD* = 14.32), with 75.5% of them being women and almost two-thirds (64.81%) of the participants having university-level qualifications. Table 1 shows the distribution of gender and level of education both in the sample and the Spanish general population.

**Table 1.** Distribution of gender and educational attainment in the sample and in the Spanish general population

		% Primary education	% Under university qualifications	% University-level qualifications	% Total
% Spanish population	Men	40.2	23.1	36.7	49
	Women	34.1	23.3	42.7	50.9
	Total	36.8	21.6	41.3	
% Sample	Men	3.3	43.9	52.8	24.5
	Women	2.4	28.9	68.7	75.5
	Total	2.59	32.53	64.81	

### Instruments

#### Problematic Use of SNs (PUS) Questionnaire

In the development of the new instrument, problematic use of SNs (PUS), we followed the criteria established by the European Federation of Psychologists’ Associations (EFPA) for test evaluation (Evers et al., 2013) and the standards for Educational and Psychological Evaluation (AERA, APA, NCME, 2014), as well as the recommendations from the current literature on psychometry (Downing & Haladyna, 2006; Lane et al., 2015; Muñiz, 2018; Muñiz & Fonseca-Pedrero, 2019).

A review of the literature was undertaken to construct a set of items representative of the behaviours that comprise problematic use of SNs (54 items). All of the items were worded directly (Suárez-Álvarez et al., 2018; Vigil-Colet et al., 2020). Problematic SN use was defined in the same way as in Marino et al. (2018a), as the use of SNs that produces negative consequences in people’s psychological well-being. From this definition, the items were developed in accordance to the two theoretical areas of problematic SN use: the addiction-related consequences of SNs use and negative social comparison. Addictive use would reflect the interference of SNs use in everyday life, while negative social comparison would reflect the comparisons made on SNs which puts the individual in a situation of inferiority. While these two facets were created to cover the theoretical aspects of maladaptive use of SNs, the internal structure of the test, however, was expected to reflect unidimensionality. This is because both addictive use and negative comparisons on SNs would both be uses of SNs with negative consequences on people’s lives. There are also meta-analyses that have examined the problematic use of SNs as a combination of the two uses (Vahedi & Zannella, 2021; Yoon et al., 2019).

A detailed qualitative and quantitative analysis was carried out to determine the representativeness of the content (Sireci & Faulkner-Bond, 2014) with the participation of 21 doctors of psychology. The expert assessment had two parts: (a) rating the clarity of the item wording on a scale from 1 to 10 and (b) rating how strongly each item belonged to each hypothesized dimension of problematic use of SNs. The expert judges were also able to comment on each item. From the data thus obtained, we applied the following three criteria to remove low-quality items: (a) inter-rater agreement below 70% with respect to the measured dimension, (b) the lower limit of the 95% confidence interval of Aiken’s V below 0.7 (Charter, 2003) and (c) unfavourable comments. Thus, we removed 34 items, with a final scale containing 20 items. The scores in the final scale for Aiken’s V were 0.88 [95% CI 0.82–0.93], indicating an excellent level of agreement about the clarity of the item wording (Penfield and Giacobbi 2004). The final instrument consisted of 20 Likert-type item with five response categories (1 completely disagree, 5 completely agree).

#### Satisfaction with Life Scale (SWLS; Diener et al., 1985)

This instrument is a scale that measures life satisfaction and it has five items. Participants are asked how much they agree with each statement, responding using a 5-point Likert-type scale (from 1 = completely disagree to 5 = completely agree). Reliability estimated using the α coefficient for the Spanish adaptation was 0.88 (Vázquez et al., 2013). The α coefficient in the present study was 0.82.

#### Hospital Anxiety and Depression Scale (HADS; Zigmond & Snaith, 1983)

We used the Spanish adaptation from Terol et al. (2007). This is a questionnaire containing 14 items, with two subscales of 7 items each, responses to which are given on a Likert-type scale from 0/3. One subscale, HADS_A, assesses the level of anxiety, and the other, HADS_D, assesses the level of depression. Higher scores in each subscale indicate greater anxiety and depression, respectively. The internal consistency of the two scales in the Spanish version was 0.86 (Quintana et al., 2003). The α coefficient in the present study was 0.84 for HADS_A and 0.77 for HADS_D.

#### Attentional Control Scale

We included an attentional control scale in order to detect participants who responded randomly to the different questionnaires. It was composed of 10 items that asked participants to choose a specific response (e.g., in this question, mark completely agree).

### Procedure

We used snowball sampling via SNs to obtain the sample. Participants had to be 18 years old or older to participate. Data collection was done through an online questionnaire, anonymously and voluntarily, with informed consent being given before starting. Both the questionnaire items and the attentional control items were presented to the participants in random order. The participants received no reward for their participation.

### Data Analysis

We used the SPSS 24 statistics package (IBM Corp, 2016) to calculate the descriptive statistics, differential item functioning (DIF), Pearson correlations and the canonical correlation. We used FACTOR 10.10.02 (Lorenzo-Seva & Ferrando, 2013) to perform the exploratory factor analysis (EFA) and to calculate reliability coefficients. Finally, we used the Mplus8 program (Muthén & Muthén, 2017) to carry out the confirmatory factor analysis (CFA).

To examine the internal structure of the test, we randomly divided the sample into two subsamples. We carried out an EFA in the first subsample, made up of 30% of the total sample (305 subjects), and a CFA in the second subsample (698 subjects). To check that the data was suitable for EFA, we used KMO and Bartlett’s statistic. The analysis was done with a polychoric correlation matrix given the ordinal nature of the variables and the high number of items with kurtosis and skewness values greater than |1| (Ferrando Piera, 2021; Muthen & Kaplan, 1992). The method of estimation was robust unweighted least squares (RULS), following the guidelines in the current literature (Lloret-Segura et al., 2014). We determined the number of factors using the optimal implementation of parallel analysis (PA) procedure (Calderón et al., 2019; Timmerman & Lorenzo-Seva, 2011).

Subsequently, in order to confirm the dimensionality indicated by the EFA, we performed a CFA with the remaining 70% of the sample. The method of estimation used was unweighted least squares estimates with standard errors and a mean- and variance-adjusted chi-square test (ULSMV). The indices of fit were *comparative fit index* (CFI), *Tucker-Lewis index* (TLI), *root mean square residual* (RMSR) and *root mean square error of approximation* (RMSEA). A good fit was observed if CFI >0.95, TLI >0.95, RMSR < 0.08 and RMSEA < 0.05 (Hu & Bentler, 1999).

Following both factor analyses, we carried out an item analysis in accordance to the classical test theory model. To assess the discrimination indices of the items, the corrected item-test correlations were calculated, with values higher than 0.2 considered acceptable (Muñiz et al., 2005; Muñiz & Fonseca-Pedrero, 2019). We also examined whether the items had an impact as a function of the variable sex. In the items that did show an impact, we assessed DIF via the logistic regression procedure (Gómez-Benito et al., 2013).

We used Cronbach’s (1951) *α* coefficient and Mcdonald’s (1999) *ω* coefficient to examine reliability.

We examined evidence of validity in relation to other variables by calculating the Pearson correlation between the new instrument and the following variables: (a) the HADS anxiety and depression scales, and (b) the Satisfaction with Life Scale (SWLS). To observe the overall relationship between problematic SNs use and psychological distress, a canonical correlation was carried out between the two dimensions found in the PUS and mood-related variables (anxiety, depression and life satisfaction). To avoid over-representation of high educational level women, all correlational analyses have been done weighting the sample to resemble to the population structure.

Internal evidence of convergent validity between the two subscales of the PUS instrument was assessed using the average variance extracted (AVE). This was done using the method described in Fornell and Larcker (1981), with values above 0.50 considered satisfactory (Hair et al., 2009). Evidence of discriminant validity between the two scales in the instrument was determined by comparing whether the AVE values for each scale were higher than the square of the correlation between them (Fornell-Larcker criterion).

In order to improve the interpretation of the results of the questionnaire, we constructed norm-referenced scores for each of the two subscales. To determine whether it was necessary to differentiate between the sexes, an independent samples weighted *t* test was performed.

## Results

We first performed an EFA with a subsample of 305 subjects. Prior to this analysis, we confirmed the suitability of the data for EFA via the KMO test (KMO = 0.87) and Bartlett’s test (*p* ≤ 0.001). An initial EFA was done to confirm whether the data fitted to an essentially unidimensional structure, which gave an unsatisfactory fit (Table 2). Despite the value of GFI and TLI being greater than 0.95, indicating an adequate fit, neither RMSEA nor RMSR gave an acceptable fit, and in addition, the PA suggested the extraction of two factors.

**Table 2.** Exploratory factor analysis of the PUS questionnaire

	Unidimensional EFA	Bidimensional EFA^1^	Bidimensional EFA^2^
GFI	0.95	0.99	0.99
TLI	0.95	0.99	0.99
RMSEA	0.10	0.05	0.06
RMSR	0.12	0.06	0.06
Explained variance	48.4%	61.3%	63.68%

*Note*. *n* = 305; *GFI* goodness of fit index; *TLI* Tucker-Lewis index; *RMSR* root mean square of residuals; *RMSEA* root mean square error of approximation; unidimensional EFA, single factor model using 20 items; bidimensional EFA^1^, two-factor model with 20 items; bidimensional EFA^2^, two-factor model using 18 items

We subsequently performed a second EFA to determine the fit of the two-dimensional structure, which produced an adequate fit with a GFI and TLI that was higher than the first EFA and an acceptable value of RMSEA and RMSR (see Table 2). However, we eliminated two items from the test (items 5 and 10) as they loaded low and equally on both factors. They were also removed for theoretical reasons as they assessed similar aspects as other items with higher factor loadings. The test was reduced to 18 items, which were analysed by a third EFA, giving adequate fit supported by the GFI and TLI being greater than 0.95, the explained variance and the RMSEA giving a value between 0.05 and 0.08 as well as the RMSR being lower than 0.08. The fit indices of all three EFAs are shown in Table 2.

The correlation between factors was 0.59. These results indicate that the test can be understood as a bidimensional scale. Based on the distribution of the items in each of the factors, the first factor could be called “negative social comparison” and the second “addictive consequences”.

The bidimensional model was tested via a CFA with 698 subjects and 18 items. Table 3 shows the values for CFI and RMSEA indicating a good fit. It also shows the factor loadings, which were very high in both factors. In addition, the table includes all the discrimination indices (DI), which were all higher than 0.56 in the first factor and higher than 0.55 in the second factor. Items 1, 12 and 17 showed impact but none of them exhibited gender DIF.

**Table 3.** Confirmatory factor analysis and discrimination index for the items in the PUS questionnaire

Items		F.L. Social comparison	F.L. Addictive consequences	DI^1^	DI^2^
1	When I see content from influencers or celebrities, I feel inferior	0.73		0.60	
2	When I see content from my friends or people I know, I feel inferior	0.81		0.74	
3	When I’m not on social networks, I feel an impulse to go online that is hard to resist		0.78		0.68
4	When I publish my content, I worry that it will be made fun of	0.68		0.62	
5	When I see what celebrities or influencers publish, I feel bad about myself	0.80		0.64	
6	When I see what my contacts publish, I feel that they have a better life than me	0.78		0.66	
7	I spend too much time using social networks		0.71		0.67
8	I don’t get enough sleep because of using social networks		0.70		0.55
9	I have tried to spend less time on social networks, but I have not been successful		0.72		0.61
10	Most of my friends and people I know on social networks are happier than I am	0.72		0.57	
11	I compare myself with other people who I think are better than me on social networks	0.89		0.73	
12	I worry that my posts will not have enough positive interactions	0.76		0.64	
13	I worry a lot about what people might think of my content	0.77		0.68	
14	I feel lonely when I see what my contacts post on social networks	0.80		0.65	
15	My academic or work performance has declined because of using social networks		0.76		0.58
16	People close to me have complained because I use social networks too much		0.71		0.59
17	I feel that if I am not connected to social networks, I am missing out on something		0.74		0.60
18	Using social networks, I lose track of time and ignore important tasks I have outstanding		0.81		0.69
CFI		0.95			
TLI		0.94			
RMSEA _(90% CI)_		0.07_(0.068–0.079)_			

*Note*. *n* = 698; *F.L.* factor loadings; *CFI* comparative fit index; *TLI* Tucker-Lewis index; *RMSR* root mean square of residuals; *RMSEA*_(90% CI)_ root mean square error of approximation (90% confidence intervals); *DI*^1^ discrimination index of the items in the first factor, called negative social comparison; *DI*^2^ discrimination index of the items in the second factor, called addictive consequences

The first PUS factor, called “negative social comparison”, had an alpha coefficient of 0.94 and an omega coefficient of 0.94. The second PUS factor, called “addictive consequences”, had an alpha coefficient of 0.91 and an omega coefficient of 0.91.

Convergent validity between scales was acceptable for both the negative social comparison factor (AVE = 0.60) and the addictive consequences factor (AVE = 0.56), with AVE values above 0.5. For discriminant validity, we compared the square of the correlation between the two factors (*r*^2^_xy_ = 0.26) with the value of AVE for each factor. As the value of AVE for both factors was greater than the square of the correlation, adequate evidence of discriminant validity between scales was found.

With regard to the relationships with other variables, Table 4 shows the Pearson correlations between problematic use of SNs, as measured using the new PUS instrument, and the variables depression, anxiety and satisfaction. The correlation between the HADS subscales (anxiety and depression) and the PUS subscales (negative comparison and addictive consequences) was statistically significant and positive. In addition, the SWLS (satisfaction) correlated negatively with both PUS subscales.

**Table 4.** Pearson correlations between the negative social comparison subscale, addictive consequences subscale, the HADS questionnaire, and the SWLS scale

	Anxiety	Depression	Satisfaction
Comparison	0.46**	0.46**	−0.35**
Addictive	0.22**	0.21**	−0.15**

**p ≤ 0.001

*Note: N* = 1003; Anxiety: the HADS subscale measuring anxiety; Depression: the HADS subscale measuring depression; Satisfaction: satisfaction with life scale; Comparison: the negative social comparison on SNs subscale from the problematic use of SNs scale (PUS); Addictive: the addictive consequences subscale from the problematic use of SNs scale (PUS)

Similarly, the canonical correlation between the two PUS subscales and the three psychological trait scales (anxiety, depression and satisfaction) was 0.52, and the redundancy coefficient was 0.16 (16% common variance).

Finally, we norm-referenced the scores based on percentiles differentiating between the sexes, as the independent sample weighted *t* test indicated statistically significant differences. Women had significantly higher scores than men in both the negative social comparison subscale (*p* = 0.003) and the addictive consequences subscale (*p* = 0.016). The norm-referenced scores for negative social comparison and addictive consequences for both men and women are shown in Table 5 and 6, respectively.

**Table 5.** Norm-referenced scores for the negative social comparison subscale divided by sex

Men		Women	
Direct score	Percentile	Direct score	Percentile
10	20	10	15
11	24	11	20
12	37	12	27
13	49	13	33
14	53	14	39
15	56	15	42
16	61	16	47
17	64	17	54
18	66	18	58
19	69	19	61
20	72	20	70
21	80	21	78
22	82	22	79
23	85	23	83
24–25	87	24	85
26	93	25	86
27	94	26	88
28	95	27	89
29	96	28	92
30	97	29	93
31	98	30	94
32–40	99	31–32	95
		33–34	96
		35	98
		36–40	99

**Table 6.** Norm-referenced scores for the addictive consequences subscale divided by sex

Men		Women	
Direct score	Percentile	Direct score	Percentile
8	14	8	17
9	16	9	23
10	21	10	30
11	30	11	37
12	32	12	42
13	36	13	47
14	39	14	53
15	47	15	57
16	56	16	61
17	60	17	67
18	72	18	75
19	75	19	76
20	78	20	82
21	79	21	86
22	86	22	88
23	88	23	90
24	90	24	92
25	92	25	93
26	97	26	96
27–30	98	27	97
31–40	99	28	98
		29	98
		30–40	99

## Discussion and Conclusions

The objective of our study was to develop and validate the problematic use of SNs (PUS) scale. The PUS is novel because (i) it is the only self-report, as far we know, that not only studies the potential addiction-related consequences of SNs use but also focuses on how SNs are used in a comparative way; and (ii) it assesses the problematic use of SNs without focusing on any specific social network, and thus, it can be generalized to different SNs.

In terms of internal structure, the new PUS scale demonstrated a bidimensional structure with two strongly correlated dimensions. Therefore, on the one hand, it is possible to obtain a score for each of the subscales for more specific information about problematic use of SNs. On the other hand, considering the high correlation of the subscales, a total problematic use score could also be obtained. One of the subscales, addictive consequences, can be considered similar to other scales that evaluate the addictive use of SNs in order to compare its structure. Most of the scales that assess this addictive use of SNs are unidimensional, such as the Social Media Disorder Scale (Van Den Eijnden et al., 2016), the Facebook Intrusion Questionnaire (FIQ) from Elphinston and Noller (2011) and the Bergen Facebook Addiction Scale (Andreassen et al., 2012) which focuses on Facebook, as well as its adaptation for SNs in general, the Bergen Social Media Addiction scale (Andreassen et al., 2016). However, there are other scales that evaluate this kind of SNs use with multidimensional factor structures, such as the TDI-RS from Chóliz et al. (2016).

The second factor (i.e. negative social comparison) is more difficult to compare, as there are no existing questionnaires that assess the same concept. This factor refers to the upward social comparison that could take place in the use of SNs and that may be detrimental to psychological well-being (Verduyn et al., 2020). Pre-existing questionnaires that have assessed social comparison on SNs are of two types: (i) the ones developed exclusively for a specific study with no intention of being validated and (ii) the ones adapted from questionnaires addressing social comparison in general which have been reformulated a fit a SNs environment. This latter group includes the questionnaire created in the 2017 study by Liu et al. in which the authors adapted a questionnaire for assessing social comparison in a day-to-day non-virtual context so that it could be used to assess upward comparisons on SNs. In the study, the authors performed a factorial analysis that showed a unidimensional structure, as did Hanna et al. (2017).

Our new PUS instrument demonstrated excellent reliability according to the European model of test quality assessment (Muñiz, 2018), both in the negative social comparison (*α* = 0.94; *ω* = 0.94) and addictive consequences (*α* = 0.91; *ω* = 0.91) subscales. The latter demonstrated greater reliability than other questionnaires that measure the addictive use of SNs, such as the short version of the Social Media Disorder Scale (Van Den Eijnden et al., 2016), the Facebook Intrusion Questionnaire (Elphinston & Noller, 2011), the Bergen Facebook Addiction Scale (Andreassen et al., 2012) and the Bergen Social Media Addiction Scale (Andreassen et al., 2016). In addition, none of the items in the PUS scale exhibited DIF based on sex, and all items had high discriminatory power. It is not possible to compare the reliability of the negative social comparison subscale with validated questionnaires that measure negative comparative use of SNs as we did not find any. However, it exhibited notably higher reliability than the “ad hoc” questionnaires used by other authors (Cramer et al., 2016; Schmuck et al., 2019; Tandoc et al., 2015).

In terms of evidence of validity related to other variables, the two PUS subscales demonstrated strong positive correlations with both anxiety and depression and a negative correlation with life satisfaction. Furthermore, the canonical correlation also indicates the relationship between these two subsets. This is consistent with previous studies that have found a relationship between the use of SNs for comparisons and anxiety and depression (Hussain & Griffiths, 2018) as well as with dissatisfaction with life (Verduyn et al., 2017). Also, it is consistent with previous studies that have found a relationship between the addictive use of SNs and anxiety and depression (Barbar et al., 2021; Keles et al., 2020; Malaeb et al., n.d.; Seabrook et al., 2016; Youssef et al., 2021) and lower levels of psychological well-being (Arias-de la Torre et al., 2020; Huang, 2017).

It is worth highlighting the differences between the PUS subscales. The negative social comparison subscale correlated more strongly with depression, anxiety and dissatisfaction with life than the addictive consequences subscale. Consequently, this may indicate the importance of evaluating the social comparison carried out in SNs in the evaluation of the problematic use of SNs in addition to its addictive consequences. This importance of social comparison, even over and above that of addictive traits, has already been highlighted by other studies (Keles et al., 2020).

The present study comes with some limitations. Firstly, the sampling was not strictly random, which means that any generalization will be limited. In addition, the sample is mainly comprised of highly qualified women. In order to address the latter, weighting to population has been calculated, but it is still a limitation to be taken into consideration. We did not obtain data from under 18 years old, which means that it might be useful to replicate this study with minors. Additionally, data was collected during the Covid-19 lockdown which may have increased problematic and non-problematic use of SNs (Brailovskaia & Margraf, 2021).

In conclusion, the present study has developed a new scale for the assessment of the problematic use of SNs. The instrument is composed of 18 items divided into two subscales: addictive consequences and negative social comparison. The instrument demonstrated excellent psychometric properties. This new tool will allow a more complete assessment of the inappropriate use of SNs by not only assessing the consequences of such use but also the tendency to use of SNs to make interpersonal comparisons. In addition, we have created two normative percentile scales differentiating between sexes in each of the two subscales.

## Declarations

The study was not explicitly reviewed by an Ethics Committee, given that this is not required by our University of Oviedo, nor by the national guidelines established in the Code of Ethics of the Spanish Psychological Association. There are several reasons why an explicit approval by an Ethics Committee was not necessary: the participants evaluated were adults, the evaluation was voluntarily accepted, that is, an implicit informed consent is assumed, and the data is treated anonymously and confidentially. In addition, all the recommendations established in ISO-10667 Standard for the evaluation of people were strictly followed. The whole evaluation process and the use of the measuring instruments were carried out always following the Deontological Code of the Spanish Psychological Association (2010), as well as the International Test Commission Guidelines for Test Use (2013).
